# Contribution to the knowledge of seed-beetles (Coleoptera, Chrysomelidae, Bruchinae) in Xinjiang, China

**DOI:** 10.3897/zookeys.466.7283

**Published:** 2014-12-18

**Authors:** You Li, Zhiliang Wang, Jianjun Guo, Jesús Romero Nápoles, Yingchao Ji, Chunyan Jiang, Runzhi Zhang

**Affiliations:** 1 Key Laboratory of Zoological Systematics and Evolution, Institute of Zoology, Chinese Academy of Sciences, Beijing 100101, China; 2 School of Forest Resources and Conservation, University of Florida, Gainesville, FL 32611, USA; 3 Institute of Entomology, Guizhou University, The Provincial Key Laboratory for Agricultural Pest Management of Mountainous Region, Guiyang 550025, China; 4 Instituto de Fitosanidad, Colegio de Postgraduados, km 36.5 carr. Fed. México-Texcoco, Montecillo, Estado de México, C.P. 56230, México; 5 College of Plant Protection, Shandong Agricultural University, Tai’an 271018, China

**Keywords:** New record, checklist, taxonomy, Palaearctic region

## Abstract

Nineteen species of seed-beetles belonging to the subfamily Bruchinae (Coleoptera, Chrysomelidae) were collected in Xinjiang, China. Of these, the following four were new records for China: *Bruchus affinis* Frolich, 1799, *Bruchus atomarius* L., 1761, *Bruchus loti* Paykull, 1800 and *Kytorhinus kergoati* Delobel & Legalov, 2009. We provide an annotated checklist, illustrations and a key to the 19 species.

## Introduction

Xinjiang Uygur Autonomous Region (hereafter referred to as Xinjiang, also known as Sinkiang) is a provincial region in the northwest of the People’s Republic of China. It is the largest Chinese administrative division and it spans over 1.6 million km^2^. The region is bordered by eight countries: Russia, Mongolia, Kazakhstan, Kyrgyzstan, Tajikistan, Afghanistan, Pakistan and India.

Four tribes of the Chrysomelidae subfamily Bruchinae have been recorded in Xinjiang: Amblycerini, Bruchini, Kytorhinini and Rhaebini (Anton 2010, Tan and Yu 1980) and six tribes according to Bouchard et al. (2011): Amblycerini, Bruchini, Eubaptini, Kytorhinini, Pachymerini and Rhaebini. Kytorhinini and Rhaebini are monotypic and restricted to Central Asia and the temperate Holarctic region, respectively (Borowiec 1987, Delobel and Legalov 2009). The majority of Bruchinae species, commonly called bean weevils or seed-beetles, feed on grain legumes and seeds of leguminous trees and shrubs. Many species have a significant economic impact because they can consume valuable protein-rich crops that would otherwise be eaten by humans (Southgate 1979). Despite this, little is currently known about the seed-beetles of Xinjiang. Fifteen species have been documented (Hoffmann 1965, Tan and Yu 1980, Zhang et al. 1987, Xu 1991, Anton 2010, Sui et al. 2011), but most of the records lack detailed information about their distribution.

## Methods

We checked all seed-beetles specimens from Xinjiang (most collected from 1956 to 1978) in National Zoological Museum of China (NZMC), Institute of Zoology, Chinese Academy of Science (IZCAS), in Beijing, China. In order to increase the material currently available in the NZMC collection, we collected twice in Xinjiang in July 2009 and August 2013. Seed-beetles were obtained in the field by sweeping with a sweep net and by collecting seeds of host plants in day time. We also tried to collect at night, but no seed-beetles were found. The identification of some of the specimens were confirmed by Chinese Chrysomelidae specialist Tan Juanjie and Yu Peiyu of IZCAS twenty years ago. All the specimens were identified by comparing the external morphological features and the male genitalia morphological characters with some published articles again (Lukjanovitsch and Ter-Minassian 1957, Tan and Yu 1980, Borowiec 1987, 1991, Kingsolver 2004, Delobel and Legalov 2009).

Photographs of all the seed-beetles were taken with a Cannon 5D digital camera and images were processed in Adobe Photoshop CS5. Drawings were created using Adobe Illustrator CS4. All specimens were deposited in the NZMC, where most of them were assigned unique numbers corresponding to the Institute of Zoology collection code entry IOZ(E).

## Results

In this study, 19 species of Bruchinae beetles were collected in Xinjiang. They were annotated with updated detailed distribution in the following checklist. The following key is illustrated with photographs of morphological characters used in it.

### Checklist of Bruchinae from Xinjiang, China

#### Tribe Amblycerini Bridwell, 1932

##### Subtribe Spermophagina Borowiec, 1987

###### Genus *Spermophagus* Schoenherr, 1833

####### Spermophagus sericeus

Taxon classificationAnimaliaColeopteraChrysomelidae

(Geoffroy, 1785)

Figs 1–2

######## Material.

2♂, Akqi, Kizilsu, Xinjiang, 40.98°N, 78.70°E, alt. ca 1970 m, 2005.VI.14, H.Y. Hu leg.; 1♀6♂, Aksu, Xinjiang, 40.94°N, 80.11°E, alt. ca 1180 m, 1978.VI.19, Y.H. Han leg.; IOZ(E)1016347–1016583; 1♀, Qinggil, Altay, Xinjiang, 46.69°N, 90.39°E, alt. ca 1390 m, 1956.VIII.1, W.Y. Yang leg., IOZ(E)632314; 1♀1♂, Turpan, Xinjiang, 42.93°N, 89.27°E, alt. ca 140 m, 1958.V.20, C.Q. Li and G. Wang leg., IOZ(E)632431, IOZ(E)632433; 3♀3♂, Baicheng, Aksu, 41.78°N, 81.92°E, alt. ca 1310 m, 1959.VII.22, A.F. Tian leg., IOZ(E)115170–115176; 1♀3♂, Yuli, Mila, Bayingol, Xinjiang, 41.77°N, 84.24°E, alt. ca 1000 m, 1958.VII.13, C.Q. Li leg., IOZ(E)115143–115146; 1♀1♂, Urumqi, Xinjiang, 43.83°N, 87.55°E, alt. ca 820 m, 1955.VII.25, S.J. Ma, K.L. Xia and Y.L.Chen leg., IOZ(E)115133–115134; 2♀2♂, Usu, Qoqek, Xinjiang, 45.02°N, 84.78°E, alt. ca 290 m, 1957.VI.16, G. Wang leg., IOZ(E)115106–115109; 10♀16♂, Shihezi, Xinjiang, 44.28°N, 86.27°E, alt. ca 500 m, 1957.VI.7, G. Wang and C.P. Hong leg., IOZ(E)115080–115106; 1♀6♂, Shawan, Qoqek, Xinjiang, 44.46°N, 85.66°E, alt. ca 420 m, 1957.VI.11, G. Wang and C.P. Hong leg., IOZ(E)115063–115169; 1♀, Jeminay, Altay, Xinjiang, 47.43°N, 85.87°E, alt. ca 970 m, 1956.IX.17, W.Y. Yang leg., IOZ(E)115054.

######## Distribution.

Widely distributed around the Palaearctic region.

**Figures 1–12.**
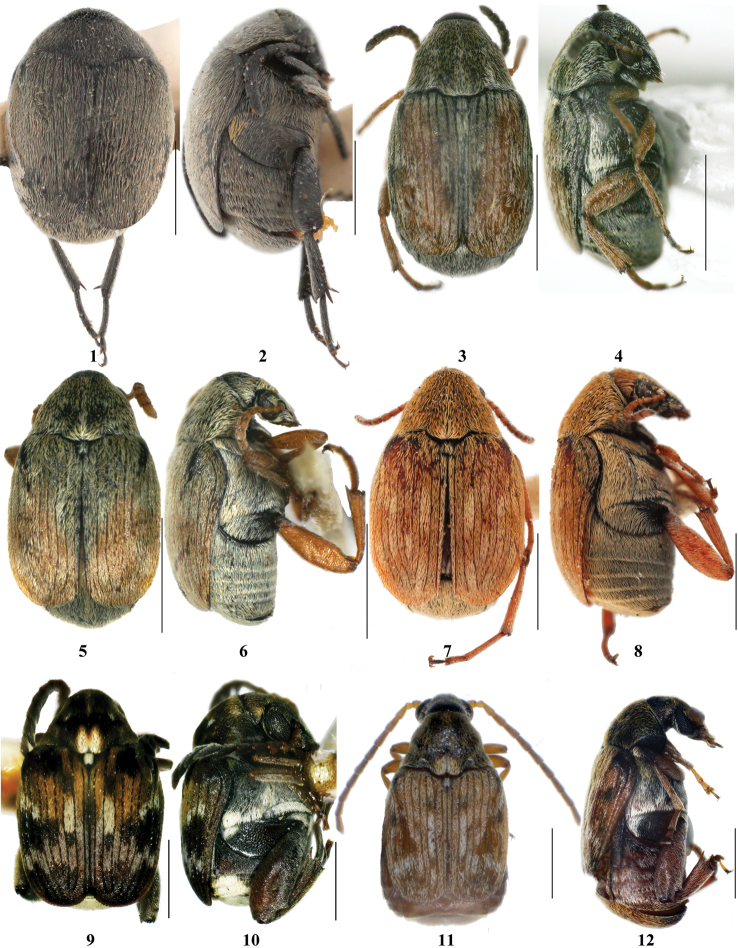
Bruchinae in Xinjiang, dorsal and lateral view. **1–2** *Spermophagus sericeus* **3–4** *Acanthoscelides pallidipennis* **5–6** *Bruchidius apicipennis* **7–8** *Bruchidius tuberculicauda* **9–10** *Callosobruchus chinensis* **11–12** *Callosobruchus maculatus*; black bar = 1 mm.

#### Tribe Bruchini Latreille, 1802

##### Subtribe Acanthoscelidina Bridwell, 1946

###### Genus *Acanthoscelides* Schilsky, 1905

####### Acanthoscelides pallidipennis

Taxon classificationAnimaliaColeopteraChrysomelidae

(Motschulsky, 1874)

Figs 3–4 , 49

######## Distribution.

Armenia, Austria, Azerbaijan, Bulgaria, China, Croatia, Czech Republic, France, Germany, Greece, Hungary, Italy, Japan, Macedonia, North America, North Korea, Russia, Slovakia, Switzerland, Serbia and Montenegro, Tajikistan.

######## Remarks.

We did not find any specimens of *Acanthoscelides pallidipennis* in Xinjiang in our to study, however Tan and Yu (1980) recorded it in Xinjiang. According to Tan and Yu (1980), the North American bruchid *Acanthoscelides pallidipennis* was introduced to China with its natural host *Amorpha fruticosa* L. a number of years ago. It has been a major pest of *Amorpha fruticosa* seeds in China.

###### Genus *Bruchidius* Schilsky, 1905

####### Bruchidius apicipennis

Taxon classificationAnimaliaColeopteraChrysomelidae

Heyden, 1892

Figs 5–6

######## Material.

2♀5♂, Korla, Bayingol, Xinjiang, 41.61°N, 86.22°E, alt. ca 1060 m 1958.VIII.11–17, C.Q. Li, IOZ(E)109480–109482, 109484, 109486, 109488–109489; 1♂, Aksu, Xinjiang, 41.18°N, 80.19°E, alt. ca 1210 m, 1958.IX.9, C.Q. Li, IOZ(E)109517; 1♂, Karakax, Hetian, Xinjiang, 37.79°N, 80.52°E, alt. ca 1250 m, 1958.V.8, C.Q. Li, IOZ(E)109516; 12♂13♀, Halajunxiang, Artux, Kizilsu, Xinjiang, 40.02°N, 76.81°E, alt. ca 1610 m, 1959.VI.22, S.Y. Wang leg., IOZ(E)109490–109514; 1♂, Yanqi, Bayingol, 41.80°N, 85.82°E, alt. ca 950 m, 1958.VIII.26, C.Q. Li, IOZ(E)109515; 1♀, Hetian, Xinjiang, 37.02°N, 79.98°E, 1955.V.20, S.J. Ma, K.L. Xia and Y.L. Chen leg., IOZ(E)109657; 2♀, Jinghe, Bortala, Xinjiang, 44.36°N, 83.15°E, alt. ca 1730 m, 1955.VIII.24, S.J. Ma, K.L. Xia and Y.L. Chen leg., IOZ(E)109518–109519; 1♀, Manas, Changji, Xinjiang, 44.54°N, 86.22°E, alt. ca 400 m, 1957.VI.9, G. Wang, IOZ(E)109521; 4♂3♀, Milan, Ruoqiang, Bayingol, Xinjiang, 39.27°N, 89.10°E, alt. ca 900 m 1960.IV.30, S.Y. Wang leg., IOZ(E)109649–109655; 2♀, Xiao Artux, Artux, Kizilsu, Xinjiang, 39.68°N, 75.67°E, alt. ca 2100 m, 1959.VI.17, S.Y. Wang leg., IOZ(E)109658–109659; 1♀, Shihutang, Manas, Changji, Xinjiang, 44.60°N, 86.09°E, alt. ca 370, 1957.VII.4, C.P. Hong leg., IOZ(E)109660; 1♂2♀, Wensu, Aksu, Xinjiang, 41.29°N, 80.21°E, alt. ca 1190, 1955.VI.9, S.J. Ma, K.L. Xia and Y.L. Chen leg., IOZ(E)109841–109843.

######## Distribution.

China, Iran, Kazakhstan, Mongolia, Russia, South Africa, Turkey, Turkmenistan.

####### Bruchidius tuberculicauda

Taxon classificationAnimaliaColeopteraChrysomelidae

Lukjanovitsch & Ter-Minassian, 1954

Figs 7–8 , 64–65

######## Material.

1♀1♂, Nilka, Ila, Xinjiang, 43.79°N, 82.50°E, 1124m,1994.VI.20, X.F. Huang leg.; 2♀1♂, Takeshikenzhen, Qinggil, Altay, Xinjiang, 46.18°N, 90.81°E, alt. ca 1110 m, 2013.VII.28, Y. Li leg..

######## Distribution.

China, Kyrgyzstan, Kazakhstan, Mongolia, Russia.

###### Genus *Callosobruchus* Pic, 1902

####### Callosobruchus chinensis

Taxon classificationAnimaliaColeopteraChrysomelidae

(L., 1975)

Figs 9–10

######## Material.

1♀, Xinhe, Xinjiang, 41.51°N, 82.50°E, alt. ca 980 m, 2000.VI.30, R.H. Lin leg.

######## Distribution.

Almost worldwide.

######## Remarks.

In this study, we found only one specimen of *Callosobruchus chinensis* in Xinjiang. Zhang et al. (1987) and Xu (1991), however, recorded *Vigna radiata* and *Vigna angularis* extensively infested by *Callosobruchus chinensis* in Kumul and Shihezi, Xinjiang.

####### Callosobruchus maculatus

Taxon classificationAnimaliaColeopteraChrysomelidae

(Fabricius, 1975)

Figs 11–12 , 50–51

######## Distribution.

Almost worldwide.

######## Remarks.

Although we did not collect any *Callosobruchus maculatus* specimens from Xinjiang in this study, Sui et al. (2001) previously recorded *Cicer arietinum* infested by *Callosobruchus maculatus* in Kashgar City, Xinjiang.

###### Genus *Megabruchidius* Borowiec, 1984

####### Megabruchidius dorsalis

Taxon classificationAnimaliaColeopteraChrysomelidae

(Fabraeus, 1839)

Figs 13–14 , 66–67

######## Material.

10♀5♂, Ili Forestry Science Research Institute, Gulja, Ili, Xinjiang, 43.94°N, 81.33°E, alt. ca 660 m, 1973.VII.5, IOZ(E)109814–109818, 632556–632565.

######## Distribution.

Bulgaria, China, France, Greece, Hong Kong, Hungary, India, Italy, Japan, Mongolia, Papua New Guinea, Switzerland, Turkmenistan.

**Figures 13–26.**
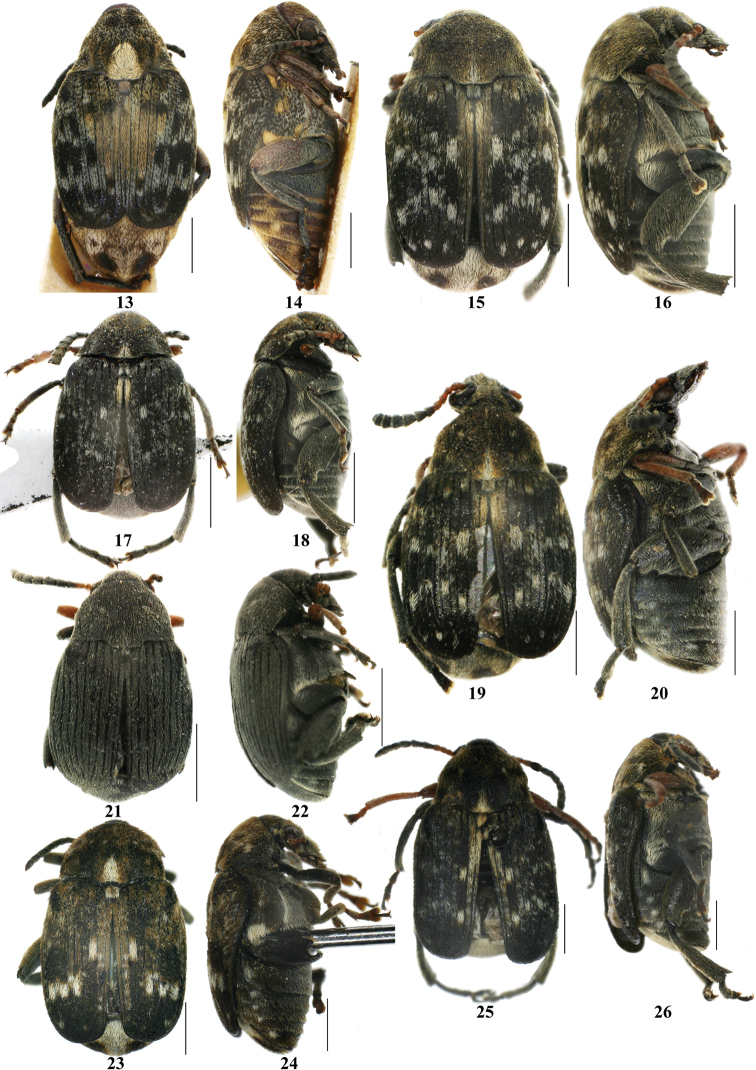
Bruchinae in Xinjiang, dorsal and lateral view. **13–14** *Megabruchidius dorsalis* **15–16** *Bruchus affinis* **17–18** *Bruchus atomarius* **19–20** *Bruchus dentipes* **21–22** *Bruchus loti* **23–24** *Bruchus pisorum* **25–26** *Bruchus rufimanus*; black bar = 1 mm.

##### Subtribe Bruchina Latreille, 1802

###### Genus *Bruchus* L., 1767

####### Bruchus affinis

Taxon classificationAnimaliaColeopteraChrysomelidae

Frolich, 1799

Figs 15–16 , 52 , 59 , 68

######## Material.

7♀3♂, Xinyuan, Ili, Xinjiang, 43.42°N, 82.26°E, alt. ca 1200 m, 1972.VII, IOZ(E)1016073–1016075, 108162–108163, 108157–108160, 108155.

######## Distribution.

China, Afghanistan, Kyrgyzstan, Kazakhstan, Lebanon, Mongolia, North Korea, Russia, Syria, Tajikistan, Europe.

####### Bruchus atomarius

Taxon classificationAnimaliaColeopteraChrysomelidae

(L., 1761)

Figs 17–18 , 53 , 63 , 68

######## Material.

5♀2♂, Xinyuan, Ili, Xinjiang, 43.42°N, 82.26°E, alt. ca 1200 m, 1972.VII, IOZ(E)1016068–1016072, 108161, 108156; 1♀, Kanasi, Buerjin County, Altay, Xinjiang, 49.01°N, 87.35°E, alt. ca 1550 m, 2009.VII.25, Z.L. Wang leg..

######## Distribution.

**New record for China**, Europe, Iran, Kyrgyzstan, Kazakhstan, Lebanon, Mongolia, North Korea, Russia, Syria.

####### Bruchus dentipes

Taxon classificationAnimaliaColeopteraChrysomelidae

Baudi, 1886

Figs 19–20 , 54

######## Distribution.

Afghanistan, Algeria, Azerbaijan, Armenia, Belgium, China, Croatia, Cyprus, Egypt, England, France, Greece, Italy, Iran, Iraq, Israel, Jordan, Kazakhstan, Lebanon, Russia, Spain, Switzerland, Syria, Tajikistan, Turkmenistan, Turkey, Uzbekistan.

######## Remarks.

We did not collect any *Bruchus dentipes* specimens in Xinjiang in this study, but Tan and Yu (1980) previously recorded *Bruchus dentipes* as occurring in Xinjiang.

####### Bruchus loti

Taxon classificationAnimaliaColeopteraChrysomelidae

Paykull, 1800

Figs 21–22 , 55 , 68

######## Material.

3♀, Xinyuan, Ili, Xinjiang, 43.42°N, 82.26°E, alt. ca 1200 m, 1972.VII, IOZ(E)1016065–1016067.

######## Distribution.

Algeria, **New record for China**, Eurasia, Japan, Morocco, Russia, Turkey, Ukraine.

####### Bruchus pisorum

Taxon classificationAnimaliaColeopteraChrysomelidae

(L., 1758)

Figs 23–24 , 56 , 60

######## Distribution.

Worldwide.

######## Remarks.

In this study, we did not find any *Bruchus pisorum* specimens in Xinjiang, but Yixin (1991) previously recorded *Bruchus pisorum* as occurring in Xinjiang.

####### Bruchus rufimanus

Taxon classificationAnimaliaColeopteraChrysomelidae

Boheman, 1833

Figs 25–26 , 46 , 57 , 62

######## Distribution.

Worldwide except Australia.

######## Remarks.

We did not find any *Bruchus rufimanus* specimens in Xinjiang; however it was previously recorded as occurring there (Tan and Yu 1980).

####### Bruchus sibiricus

Taxon classificationAnimaliaColeopteraChrysomelidae

Germar, 1824

**Figures 27–38.**
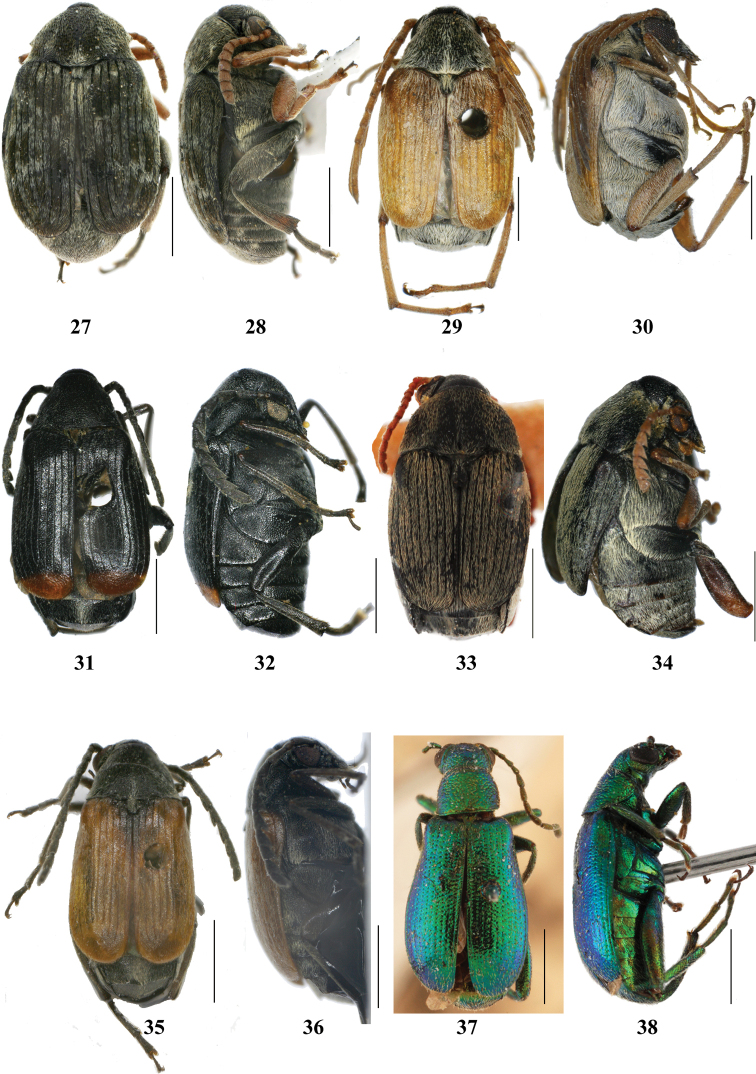
Bruchinae in Xinjiang, dorsal and lateral view. **27–28** *Bruchus sibiricus* **29–30** *Kytorhinus immixtus* **31–32** *Kytorhinus karasini* **33–34** *Kytorhinus thermopsis* **35–36** *Kytorhinus kergoati* **37–38** *Rhaebus solskyi*; black bar = 1 mm.

Figs 27–28 , 58 , 61

######## Material.

1♀1♂, North of Tianshan Mountain, Wuku Road, Urumqi, Xinjiang, 43.56°N, 87.19°E, alt. ca 1600 m, 1960.VI.11, S.Y. Wang leg., IOZ(E)1045200–1045201.

######## Distribution.

Azerbaijan, Armenia, China, Kyrgyzstan, Kazakhstan, Mongolia, Russia, Tajikistan, Turkey, Uzbekistan.

#### Tribe Kytorhinini Bridwell, 1932

##### Genus *Kytorhinus* Fischer von Waldheim, 1809

###### Kytorhinus immixtus

Taxon classificationAnimaliaColeopteraChrysomelidae

Motschulsky, 1874

Figs 29–30 , 43

####### Material.

1♂, Pochengzi, Wensu, Aksu, Xinjiang, 41.77°N, 80.99°E, alt. ca 2000 m, 1978.VI.15.

####### Distribution.

China, Kyrgyzstan, Russia.

**Figures 39–42.**
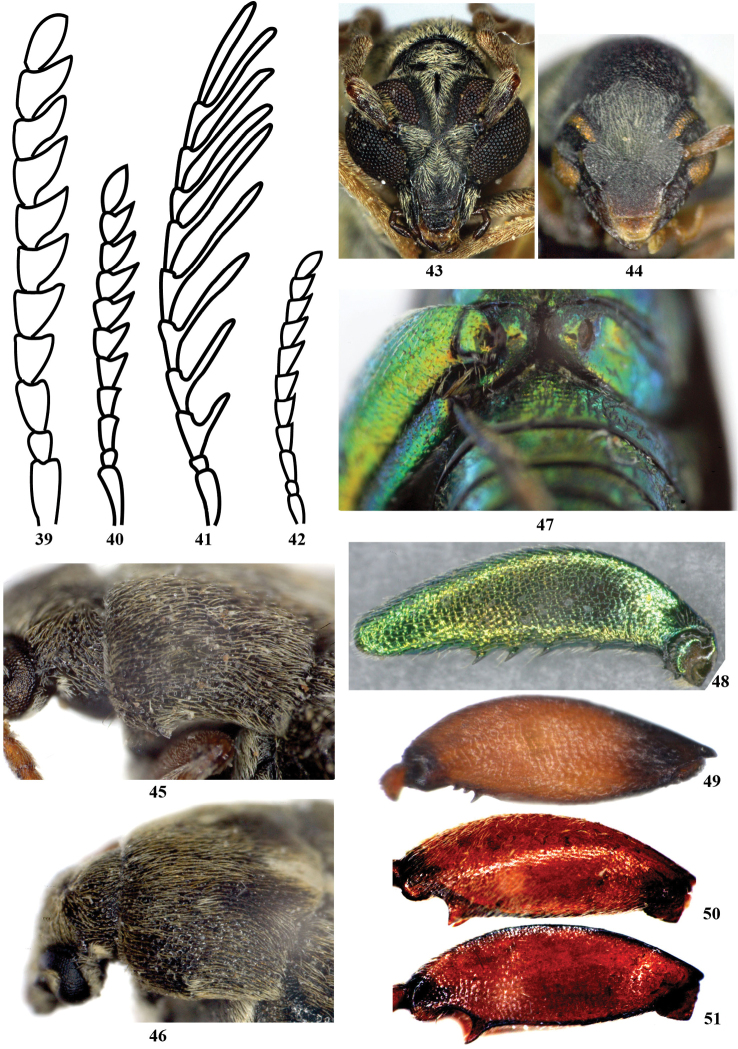
Antennae. **39** *Kytorhinus thermopsis* male **40** *Kytorhinus thermopsis* female **41** *Kytorhinus kergoati* male **42** *Kytorhinus kergoati* female **43–44** Head **43** *Kytorhinus immixtus* **44** *Kytorhinus thermopsis* **45–46** Lateral pronotal margin. **45** *Bruchus affinis* 46 *Bruchus rufimanus* **47** Hind trochanters **47** *Rhaebus solskyi* **48–51** Hind femur. **48** *Rhaebus solskyi* male **49** *Acanthoscelides pallidipennis* **50** *Callosobruchus maculatus* outside view **51** *Callosobruchus maculates* inside view.

###### Kytorhinus karasini

Taxon classificationAnimaliaColeopteraChrysomelidae

Fischer, 1809

Figs 31–32

####### Material.

1♀, Tianshan Mountain, Fukang, Changji, Xinjiang, 43.95°N, 88.15°E, alt. ca 2150 m, 1957.VII.14, G. Wang leg., IOZ(E)115178; 1♀, Tianshan Mountain, Fukang, Changji, Xinjiang, 43.95°N, 88.40°E, alt. ca 2250 m, 1957.VII.14, G. Wang leg., IOZ(E)107522; 1♀, Zhao Su Xian, Yili, Xinjiang, 42.66°N, 80.18°E, alt. ca 2200 m, 1978.VIII.7, Y.H. Han leg., IOZ(E)1045574; 1♀, Takeshikenzhen, Qinggil, Altay, Xinjiang, 46.18°N, 90.81°E, alt. ca 1110 m, 2013.VII.28, Y. Li leg.

####### Distribution.

China, Russia.

###### Kytorhinus kergoati

Taxon classificationAnimaliaColeopteraChrysomelidae

Delobel & Legalov, 2009

Figs 35–36 , 41–42 , 68

####### Material.

1♀, Pochengzi, Wensu, Aksu, Xinjiang, 41.77°N, 80.91°E, alt. ca 1930 m, 1978.VI.3, X.Z. Zhang leg., IOZ(E)1045598; 1♀1♂, Tomur peak, Wensu, Aksu, Xinjiang, 41.74°N, 80.58°E, alt. ca 2400 m, 1977.VI.19, C.J. Li leg., IOZ(E)632265–632266; 1♂, Tomur peak, Wensu, Aksu, Xinjiang, 41.81°N, 80.59°E, alt. ca 3200 m, 1977.VII.11, Y.H. Han leg., IOZ(E)632264; 1♂, Tomur peak, Wensu, Aksu, Xinjiang, 41.81°N, 80.59°E, alt. ca 3200 m, 1977.VII.14, C.J. Li leg., IOZ(E)632263; 1♂, Yangbajingzhen, Damxung, Lhasa, Tibet, China, 30.02°N, 90.39°E, alt. ca 4310 m, 1960.VI.2, C.G. Wang leg., IOZ(E)1016165.

####### Distribution.

**New record for China**, Tajikistan (Gorno-Badakhshan autonomous province).

####### Host.

Unknown.

####### Remarks.

Delobel and Legalov (2009) described this species based on a single male. We examined a female specimen and found that its antennae are serrated and are slightly longer than half of the body length (Fig. 35, 41, 42). Additionally, the elytra of the female are almost yellow, with the exception of a black, elongate triangular area, which extends from the base to one-third of the elytral suture (Fig. 35) corresponding to main distinguishing characters of a single known male of *Kytorhinus kergoati*.

###### Kytorhinus thermopsis

Taxon classificationAnimaliaColeopteraChrysomelidae

Motschulsky, 1874

Figs 33–34 , 39 , 40

####### Material.

1♀, Xinjiang, IOZ(E)115177; 1♀, 6 km northwest of Fuyun, Altay, Xinjiang, 47.14°N, 87.55°E, alt. ca 650 m, 2009.VII.13, X.L. Huang leg.

####### Distribution.

China, Kazakhstan, Mongolia, Russia.

#### Tribe Rhaebini Blanchard, 1845

##### Genus *Rhaebus* Fischer von Waldheim, 1824

###### Rhaebus solskyi

Taxon classificationAnimaliaColeopteraChrysomelidae

Kraatz, 1879

Figs 37–38 , 47–48

####### Material.

2♀1♂, Haiziwan Reservo, Shawan, Qoqek, Xinjiang, 44.56°N, 85.78°E, alt. ca 390 m, 1957.VI.9, C.P. Hong leg., IOZ(E)107501–107503; 1♀, Takeshikenzhen, Qinggil, Altay, Xinjiang, 46.18°N, 90.81°E, alt.1110 m, 2013.VII.28, Y. Li leg.

####### Distribution.

China, Kazakhstan, Mongolia, Russia.

### Key to species of Bruchinae in Xinjiang

**Table:** 

1	Body completely metallic in color (Fig. 37); hind trochanters extremely enlarged (Fig. 47); hind femur with 3–8 small, evenly spaced spines on ventral side (Fig. 48)	***Rhaebus solskyi***
–	Body not metallic in color; hind trochanters small; hind femur without 3–8 small, evenly spaced spines on ventral side	**2**
2	Antennae sexually dimorphic, male antennae strongly serrate (Fig. 39) or pectinate (Fig. 41), female antennae moderately serrate (Figs 40, 42); 3 last abdominal tergites (including pygidium) exposed behind the elytra (Figs 29, 31, 33, 35)	**3**
–	Antennae not sexually dimorphic, sometimes male and female antennae with different color; only pygidium exposed behind the elytra (Figs 62–67)	**6**
3	Elytra integument with single color (Figs 29, 33)	**4**
–	Elytra integument with two colors (Figs 31, 35)	**5**
4	Antennae, legs and elytra integument yellow (Fig. 29); eyes large and separated by 0.2 times head width including eyes (Fig. 43)	***Kytorhinus immixtus***
–	Antennae and legs reddish brown, elytra integument black (Fig. 33); eyes medium-sized and separated by 0.4 times head width including eyes (Fig. 44)	***Kytorhinus thermopsis***
5	Antennae and legs black, elytra integument almost yellow except by black basal area elongated, triangular (Fig. 35)	***Kytorhinus kergoati***
–	Body almost black, only apex of elytra red (Fig. 31)	***Kytorhinus karasini***
6	Lateral pronotal margins with tubercle (maybe obscured by setae) (Figs 45, 46); mesotibia at apex in male with apical spines or figs (Figs 52–58)	**7**
–	Lateral pronotal margins smooth without tubercle; mesotibia at apex in male without apical spines or figs	**13**
7	Elytra without white or brown setae; body almost black, only 4 basal antennal segments and fore legs reddish orange	***Bruchus loti***
–	Elytra with white or brown setae (Figs 15–28); body not almost black	**8**
8	Metatibia with mucro longer than lateral denticle (Figs 60, 61)	**9**
–	Metatibia with mucro shorter than lateral denticle (Fig. 59)	**10**
9	Four basal antennomeres, protibia and tarsi, part or all of mesotibia, and tarsi reddish orange; hind femur with long external tooth near apex; mesotibia at apex in male as Fig. 56	***Bruchus pisorum***
–	Five basal antennomeres reddish orange and rest black in female, antenna all reddish orange in male; hind femur with blunt external tooth near apex; mesotibia at apex in male as Fig. 58	***Bruchus sibiricus***
10	Pygidium with white or brown, dense and long setae, with 2 subapical black spots (sometimes subapical spots indistinct as in *Bruchus rufimanus*) (Fig. 62); mesotibia at apex in male as Fig. 52, 54 or 57	**11**
–	Pygidium with gray, sparse and short setae, without subapical spot (Fig. 63); mesotibia at apex in male as Fig. 53	***Bruchus atomarius***
11	Lateral pronotal margin with denticle at midpoint (Fig. 46); mesotibia at apex in male as Fig. 54 or 57	**12**
–	Lateral pronotal margin with denticle at 1/3 near apex (Fig. 45); mesotibia at apex in male as Fig. 52	***Bruchus affinis***
12	Lateral pronotal margin with prominent and sharp denticle; elytra with 3 rows of white stripes; pygidium with 2 distinct black subapical spots; mesotibia at apex in male as Fig. 54	***Bruchus dentipes***
–	Lateral pronotal margin with blunt denticle; elytra varying from pattern of white spots on black background with short, yellowish brown stripes to variably distributed white spots; pygidium with 2 indistinct black subapical spots (Fig. 62); mesotibia at apex in male as Fig. 57	***Bruchus rufimanus***
13	Body ovate; metatibia with 2 conspicuous apical spurs (Fig. 2); pronotum lateral margins complete	***Spermophagus sericeus***
–	Body suboval; metatibia without apical spur; pronotum lateral margins absent in apical half at least	**14**
14	Hind femur with teeth both on inner and outer margins of ventral sulci (Figs 50, 51), sometimes denticle on distal margin blunt; posterior margin of pronotum bilobed at junction with scutellum and feebly gibbose there (Figs 9, 11)	**15**
–	Hind femur with outer margin of ventral sulci edentate; posterior margin of pronotum without feeble gibbose there	**16**
15	Elytral striae 3 and 4 each with prominent subbasal denticles on slight gibbose, antennae serrate in male	***Callosobruchus chinensis***
–	Elytral striae extending to basal margin without prominent denticles or gibbose, antennae smooth linear in male	***Callosobruchus maculatus***
16	Hind femur ventrally with 3 distinct preapical teeth on inner margins of ventral sulci, proximal tooth much larger than others (Fig. 49)	***Acanthoscelides pallidipennis***
–	Hind femur ventrally edentate or with 1 preapical tooth in internal margins of ventral sulci	**17**
17	Pygidium immaculate in male (Fig. 66) and with a pair of dark patches in female (Fig. 67)	***Megabruchidius dorsalis***
–	Pygidium without patches	**18**
18	Pronotum covered with orange pubescence (Fig. 7); pygidium tuberculate in female and smooth in male (Figs 64, 65)	***Bruchidius tuberculicauda***
–	Pronotum covered with white pubescence (Fig. 5); pygidium not tuberculate	***Bruchidius apicipennis***

**Figures 52–68.**
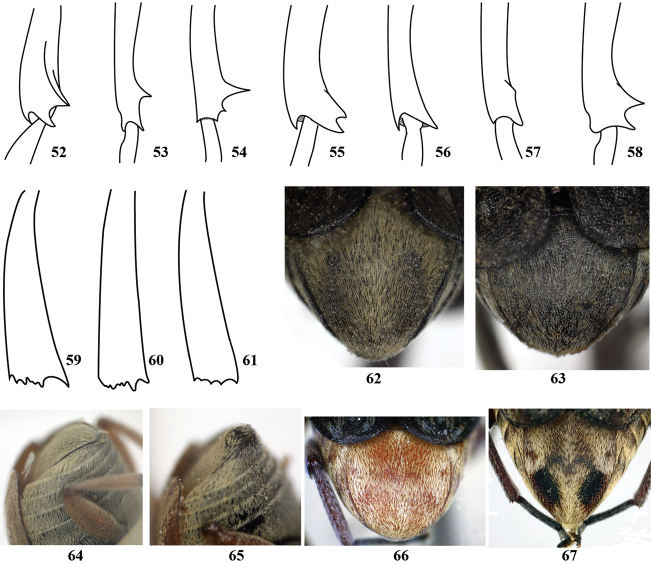
Tibia apical portion of *Bruchus* spp. male mid legs. **52** *Bruchus affinis* **53** *Bruchus atomarius* **54** *Bruchus dentipes* **55** *Bruchus loti* **56** *Bruchus pisorum* **57** *Bruchus rufimanus* **58** *Bruchus sibiricus* **59–61** Tibia apical portion of *Bruchus* spp. hind legs **59** *Bruchus affinis* **60** *Bruchus pisorum* **61** *Bruchus sibiricus* **62–67** Pygidium. **62** *Bruchus rufimanus* **63** *Bruchus atomarius* **64** *Bruchidius tuberculicauda* male **65** *Bruchidius tuberculicauda* female **66** *Megabruchidius dorsalis* male **67** *Megabruchidius dorsalis* female.

**Figure 68.**
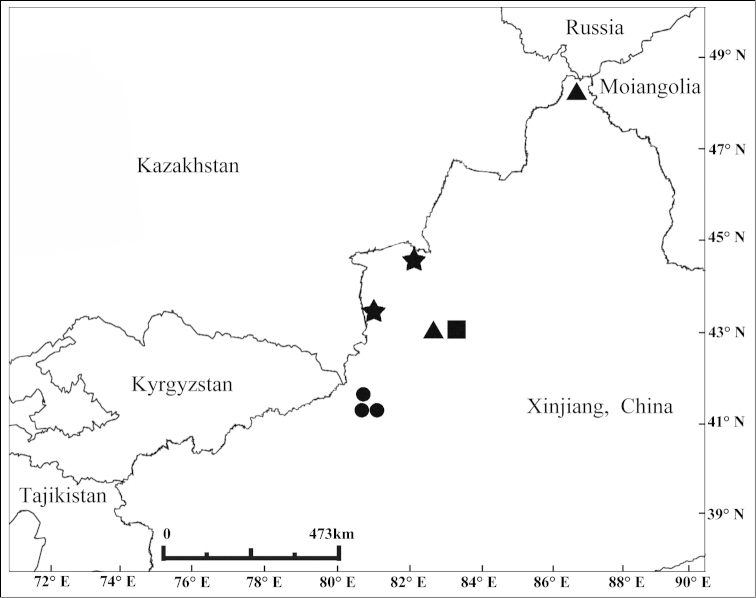
Map of northwestern China, illustrating localities for Bruchinae species. *Bruchus affinis* and *Bruchus loti* = squares. *Bruchus atomarius* = triangles. *Kytorhinus kergoati* = dots. Only new records reported here are shown.

## Discussion

Chinese literature on Bruchinae is out-of-date because of the recent changes in generic and tribal classification and description of new species (Borowiec 1987, Bouchard et al. 2011, Delobel and Legalov 2009). The majority of seed-beetle species in Xinjiang belong to the genera *Bruchus* and *Kytorhinus*. Of these, four species in this study are new records for China. These are *Bruchus affinis*, *Bruchus atomarius*, *Bruchus loti* and *Kytorhinus kergoati*. The first three have a wide distribution in the Palearctic Region. Only *Kytorhinus kergoati* has been recorded in Tajikistan. Most of the new Bruchinae distribution records are found near the border. *Acanthoscelides pallidipennis*, *Callosobruchus chinensis* and *Callosobruchus maculatus* are adventive species, so the extended human activity in Xinjiang is probably responsible for the beetle’s extended distribution in this area too. *Bruchidius apicipennis*, *Bruchidius tuberculicauda*, *Megabruchidius dorsalis*, *Rhaebus solskyi* and *Spermophagus sericeus* appear to be eurytopic species found in a wide variety of habitats of the Palearctic Region.

The Bruchinae of Xinjiang remain relatively poorly investigated and it is likely that many additional species can still be found in the region. Further fieldwork is required to ascertain if the paucity of data is due to a genuinely small number of species, or the result of insufficient collection efforts.

## Supplementary Material

XML Treatment for Spermophagus sericeus

XML Treatment for Acanthoscelides pallidipennis

XML Treatment for Bruchidius apicipennis

XML Treatment for Bruchidius tuberculicauda

XML Treatment for Callosobruchus chinensis

XML Treatment for Callosobruchus maculatus

XML Treatment for Megabruchidius dorsalis

XML Treatment for Bruchus affinis

XML Treatment for Bruchus atomarius

XML Treatment for Bruchus dentipes

XML Treatment for Bruchus loti

XML Treatment for Bruchus pisorum

XML Treatment for Bruchus rufimanus

XML Treatment for Bruchus sibiricus

XML Treatment for Kytorhinus immixtus

XML Treatment for Kytorhinus karasini

XML Treatment for Kytorhinus kergoati

XML Treatment for Kytorhinus thermopsis

XML Treatment for Rhaebus solskyi
